# ACTH-producing small cell neuroendocrine carcinoma from the gallbladder: a case report and literature review

**DOI:** 10.3389/fendo.2023.1224381

**Published:** 2023-09-12

**Authors:** Xiaofang Zhang, Dihua Huang, Xiaojie Pan, Qiya Si, Qiaoying You

**Affiliations:** Department of Endocrinology, Shaoxing People’s Hospital, Shaoxing, Zhejiang, China

**Keywords:** Cushing syndrome, ectopic adrenocorticotropic hormone syndrome, neuroendocrine tumor, gallbladder, case report

## Abstract

Ectopic adrenocorticotropic hormone syndrome (EAS) is a condition of hypercortisolism caused by non-pituitary tumors that secrete adrenocorticotropic hormone (ACTH). A rare occurrence of this syndrome is due to an ACTH-producing neuroendocrine tumor that originates from the gallbladder. One patient with severe hypokalemia and alkalosis was admitted to our hospital. Clinical presentations and radiographic findings confirmed the diagnosis of an aggressive ACTH-producing gallbladder malignancy with multiple liver metastases. The diagnosis was verified by pathological and immunohistochemical measurements from a biopsy of the hepatic metastasis. A literature review identified only four similar cases had been reported. Despite being rare and having a poor prognosis, hormone-producing neuroendocrine tumors that derive from the gallbladder should be considered in the differential diagnosis of ectopic ACTH syndrome.

## Introduction

Neuroendocrine neoplasms (NENs) are a heterogeneous group of tumors originating from neuroendocrine cells and are able to secrete amines or peptides as their neurotransmitters, such as 5-hydroxytryptamine, vasoactive polypeptide, insulin, growth hormone, adrenocorticotropic hormone, gastrin, somatostatin pancreatic polypeptide, and calcitonin. Ectopic adrenocorticotropic hormone syndrome (EAS) develops as a result of neuroendocrine tumors outside of the pituitary gland, which secrete either adrenocorticotropic hormone (ACTH) and/or corticotropin-releasing hormone, leading to a clinical presentation that resembles Cushing disease, characterized by hirsutism, muscular wasting, truncal-central obesity, hypertension, diabetes mellitus, and osteoporosis (1). The most common cause of ectopic ACTH is neuroendocrine tumors derived from the lung and anterior mediastinum. According to the largest published series (involving 383 EAS patients), lung NETs are the most common neoplasm (25%), followed by small-cell lung cancer (SCLC) (20%). Other common tumors are thymic (11%) and pancreatic NETs (8%), medullary thyroid carcinoma (6%), and pheochromocytoma (5%) (2). Tumors originating from the gallbladder and biliary duct were rarely reported.

We reported a case of severe hypokalemia and alkalosis caused by an aggressive ACTH-secreting gallbladder malignancy with numerous liver metastases. Only four comparable cases have been reported (3–6), according to a comprehensive literature review.

## Case report

A 65-year-old man was admitted to our hospital with complaints of progressive weakness and anorexia that had persisted for ten days. Prior to this, the patient had been in a normal state. His condition began to deteriorate rapidly, as he claimed to have experienced accelerated fatigue, decreased appetite, and a weight loss of 1 kg. The patient denied experiencing any abdominal pain or diarrhea. His previous medical history included hypertension for the past 8 years and type 2 diabetes mellitus for the past 5 years. Glycemia was well controlled with insulin Aspart30 during the past two years, but it has deteriorated in the last month. Additionally, he underwent a bladder mass resection surgery in 2015, but the pathology of the mass could not be traced. He had a history of smoking for over 20 years and alcohol consumption for 30 years (100 ml per day). There was also a positive family history of hypertension and diabetes mellitus. Physical examination indicated that the patient had facial blushing, central obesity with thin extremities, and proximal muscle wasting. Pitting edema was also observed in both lower limbs. Skin hyperpigmentation was not obvious. His height measured at 159 cm, weight at 64 kg, and his blood pressure at 162/102 mmHg.

**Figure 1:**
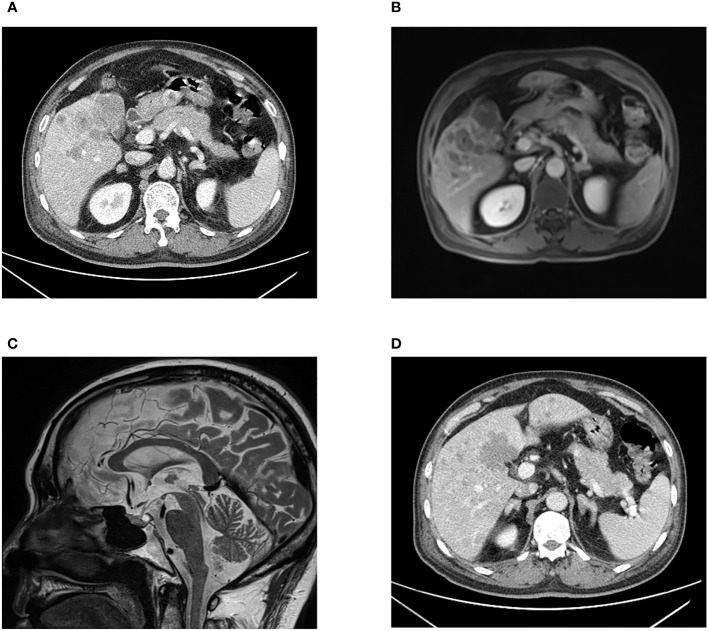
CT scan **(A)** and MRI scan **(B)** images presenting an irregular shaped mass in the gallbladder fossa, and multiple intrahepatic nodular lesions. Pituitary MRI **(C)** showing a Rathke cyst in the posterior pituitary gland with high signal intensity on T2WI. Bilaterally enlarged adrenal glands are displayed **(D)**.

**Figure 2:**
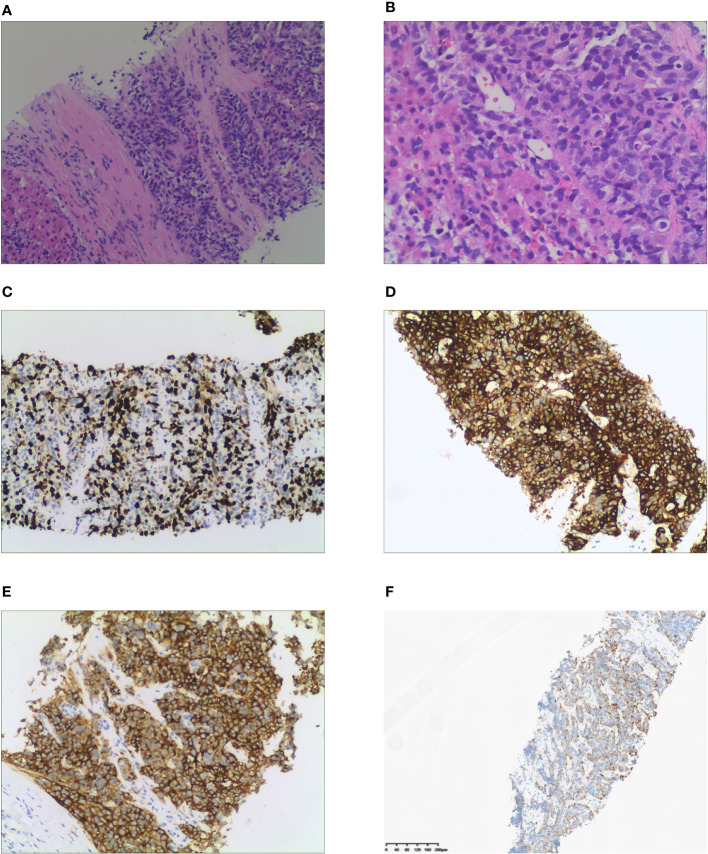
Histological and immunohistochemical examination of liver metastasis. **(A)** Tumor cells gather into nests or sheets and infiltrate the fibrous stroma (H&E stain, x100). **(B)** Cellularity is very high with hyperchromatic nuclei and no discernible nucleoli is observed (x200). **(C)** Immunohistochemical staining of Ki 67. Tumor cells demonstrate a Ki 67 index of 67%. **(D)** Tumor cells demonstrate positive expression of chromogranin A. **(E)** Positive expression of synaptophysin is observed in tumor cells. **(F)** ACTH is diffusively expressed in the cytoplasm of tumor cells.

Laboratory tests showed that the hematocrit and leukocyte count were normal, the platelet count decreased to 62 ×10^9^/L. Liver function manifested slightly elevated GGT (81.2 U/L) and total bilirubin (31.3 μmol/L). Hypoproteinemia was observed (serum albumin: 31.7 g/L). HbA1c was 7.5%, indicating that blood glucose levels had not been well controlled for the past 3 months. Notable analysis revealed a hypokalemia of 1.98 mmol/L and metabolic alkalosis. Urinary potassium excretion (138.53 mmol/24 h) markedly increased. Additional tests revealed that the patient had disrupted circadian biorhythms of plasma ACTH and cortisol (as shown in **Table 1**). The plasma ACTH level at 8 a.m. was highly elevated at 820 pg/mL (N 7-46 pg/mL). Both serum and 24-hour urine cortisol levels were remarkably beyond the upper limit of detection, and could not be suppressed by high-dose dexamethasone (administered orally, 2 mg every 6 hours for 2 days). Tumor markers, especially CEA, CA199, and AFP, were elevated. All these data raised suspicion of ACTH-dependent Cushing syndrome, and further imaging examinations were conducted in an attempt to locate the tumors. Enhanced CT (**Figure 1A**) and MRI (**Figure 1B**) scans consistently detected a mass in the gallbladder invading the liver. Multiple metastases were discovered in the liver. The pituitary gland appeared normal except for a Rathke cyst (**Figure 1C**). Bilateral adrenal hyperplasia, possibly caused by elevated ACTH, was also noted (**Figure 1D**). A CT-guided transdermal biopsy of liver metastasis was successfully performed. Immunohistochemical analysis revealed a small cell neuroendocrine tumor with positive staining of chromogranin A, synaptophysin, and ACTH (**Figure 2**) (7). Based on the evidence, a diagnosis of an ectopic ACTH-production tumor with hepatic metastases derived from the gallbladder was made.

**Table 1:** The main laboratory results in the patient with gallbladder EAS.

Items	Result	Normal range
Serum cortisol (nmol/L, 8 a.m.)	>1380	138-690
Serum cortisol (nmol/L, 0 a.m.)	>1380	138-690
ACTH (pg/ml, 8 a.m.)	820	0-46
24h-UFC (nmol)	>5114	157-645
Serum cortisol after large dose dexamethasone test (nmol/L, 8 a.m.)	>1380	≤138
CEA (ng/ml)	22.9	0-5
APF (ng/ml)	52.73	0-13.4
CA199 (U/ml)	844.19	0-37

As for the treatment, the patient received a daily dosage of 180 mg spironolactone (divided into three times) and 134 mmol of potassium. This eventually helped to maintain the serum potassium level at 3.7 mmol/L. Radical resection of gallbladder malignancy and hepatic metastases was unattainable on account of the patient’s delicate condition. Chemotherapy, molecular targeted therapy, somatostatin analogues, and peptide receptor radionuclide therapy were openly discussed with the patient and his family members. The patient initially declined these therapeutic measures. He died of advanced gallbladder malignancy, liver function failure, malnutrition, and chronic gastrointestinal bleeding, hypoxemia after one month.

Literature review found that 4 cases of gallbladder EAS were reported. Three patients had gallbladder malignancy, and one patient had cholangiocarcinoma. The main clinical characteristics are listed in **Table 2**.

**Table 2:** Ectopic ACTH syndrome caused by malignancy from the gallbladder and biliary: patient details, pathology, and tumor location.

Case Number	Sex/Age(year)	Symptom and sign	K+ (mmol/l)	ACTH (8 a.m.) (pg/ml)	Cortisol (8 a.m.) (nmol/L)	24h-UFC (nmol)	Pathology	Management and follow up	Tumor location	Reference
P1	F/44	depression hysteria, weight gain, weakness, oedema, facial plethora, hirsutism, striae and bruise, quadriceps wasting	2.5	161 (N 10-46)	137.48 (N 270-540)	ND.	poorly differentiated anaplastic adenocarcinoma	bilateral adrenalectomy; died in three months;	in the gallbladder, with a secondary tumor in the liver	(3)
P2	F/68	Not given	2.1	1459	2500	ND.	atypical carcinoid	metyrapone; died;	in the gallbladder with liver metastasis	(4)
P3	F/61	edema of the lower extremities, weight gain, moon face, truncal fat deposition, hirsutism, skin hyperpigmentation	2.1	1340	>1380	> 900	liver adenocarcinoma of bile duct origin	cholecystostomy; died one month after surgery	in the bile duct with metastatic deposit in liver	(5)
P4	F/65	weakness, anorexia, proximal muscle weakness hirsutism, moon face, buffalo hump	1.8	224	Not given	Not given	ACTH-producing large cell neuroendocrine carcinoma	cholecystectomy and wedge-shaped liver resection, followed by arterial embolization; died;	in the gallbladder	(6)

Conversion for plasma cortisol: 1 μg/dl = 27.64 nmol/l; ND, not done.

## Discussion

In this patient, elevated ACTH and serum cortisol levels supported the diagnosis of ACTH-dependent Cushing syndrome. The next challenge is to identify the tumor responsible for producing ACTH. High doses of dexamethasone may partially or completely suppress ACTH secretion for most pituitary corticotrophin tumors but not for most ectopic ACTH-secreting tumors (8). However, the high-dose dexamethasone suppression test (HDDST) is considered to have relatively low diagnostic accuracy (9). In some well-differentiated neuroendocrine tumor cases (in particular bronchial, thymic, and pancreatic carcinoids), ACTH secretion can be suppressed by high doses of dexamethasone (10). IPSS (inferior petrosal sinus sampling) is the gold standard to reliably differentiate ectopic ACTH syndrome from pituitary ACTH adenoma. For some EAS patients with indolent tumors, 68Ga-DOTATATE can be used as a tracer in PET imaging to detect criminal tumors (11). For our patients, the diagnosis of ectopic ACTH syndrome caused by a gallbladder mass could be established based on the following evidence: Firstly, high-dose dexamethasone could not suppress serum and urine cortisol levels, indicating the presence of ectopic ACTH-producing tumors. Secondly, a normal pituitary MR image ruled out the existence of an ACTH macroadenoma. Thirdly, biopsy of the hepatic metastasis revealed positive ACTH staining.

According to the new WHO classification, neuroendocrine carcinomas (NECs) are defined as >10 mitoses per 2 mm^2^ and Ki-67 >20% (often associated with a Ki-67 >55%). The carcinomas are further subtyped as small cell and large cell neuroendocrine carcinomas based on cytomorphological characteristics (12). In this patient’s case, a diagnosis of EAS led to the identification of an ACTH-producing neuroendocrine tumor derived from the gallbladder with multiple intra-hepatic metastases. Pathological and immunohistochemical examination showed positive ACTH in liver metastases with a Ki-67 of 67%, confirming the diagnosis of small cell neuroendocrine carcinoma. His general condition deteriorated rapidly and he had a poor outcome, which is consistent with the typical presentation of carcinoma.

Medical management of EAS is a complex matter. The aim is to reduce excessive cortisol levels and eliminate neuroendocrine tumors. The optimal treatment strategy is complete surgical resection of the tumor. However, most patients are at a late stage of disease when they get a final diagnosis, and only 10–30% of them have a chance for curative resection of the tumors. A combination of active chemotherapy, radiotherapy, targeted therapy, somatostatin analogues, and other multimodal treatments should be considered to minimize tumor size and prolong survival time (13). When the tumor is unresectable, management to reduce hypercortisolism should be conducted, including pharmacological agents to suppression of cortisone production or bilateral adrenalectomy. In this case, a multidisciplinary team was organized to deliberate on treatment strategies. Surgical removal of the gallbladder mass and hepatic metastases seemed impossible. Unfortunately, the patient died one month later without attempting any other therapeutic choices due to the rapid progression and deterioration of the disease.

We systematically reviewed the previous reports on ACTH-secreting gallbladder endocrine tumors. Four cases had been reported at present (3–6). All were female patients with severe hypokalemia. The neoplasms presented with highly aggressive atypical carcinoids and produced remarkably high level of ACTH and cortisol. One located in the bile duct, and the other three located in the gallbladder. Three cases had liver metastasis. All patients had rapid progression and a dismal prognosis. In our case, ACTH-producing gallbladder neuroendocrine carcinoma was confirmed by a positive ACTH immunocytochemical stain in biopsy from hepatic metastasis, which provides a novel and simple way to diagnosis.

In conclusion, ACTH-producing neuroendocrine carcinomas located in the gallbladder are rare. We present a case of a male patient with a gallbladder NEC that secretes ACTH. The disease advanced rapidly and had a poor prognosis. Recognition of its clinical condition by laboratory measurements, radiological and immunohistochemical examinations may benefit in an earlier diagnosis and a better chance of life-saving management.

## Data availability statement

The original contributions presented in the study are included in the article/supplementary material. Further inquiries can be directed to the corresponding author.

## Ethics statement

The studies involving humans were approved by the ethics committee of Shaoxing People’s Hospital. The studies were conducted in accordance with the local legislation and institutional requirements. The participants provided their written informed consent to participate in this study. Written informed consent was obtained from the individual(s) for the publication of any potentially identifiable images or data included in this article.

## Author contributions

XZ and DH are co-first authors of this case report. XZ conducted the writing and literature search. DH performed the acquisition, analysis and interpretation. XP and QS carried out the medical practice. QY is the corresponding author supervising this work. All authors contributed to the article and have approved the submitted version.

## References

[1] Isidori AM Kaltsas GA Pozza C Frajese V Newell-Price J Reznek RH . The ectopic adrenocorticotropin syndrome: clinical features, diagnosis, management, and longterm follow-up. J Clin Endocrinol Metab (2006) 91(2):371–7. doi: 10.1210/jc.2005-1542 16303835

[2] Isidori AM Lenzi A . Ectopic ACTH syndrome. Arq Bras Endocrinol Metabol (2007) 51(8):1217–25. doi: 10.1590/s0004-27302007000800007 18209859

[3] Spence RW Burns-Cox CJ . ACTH-secreting 'apudoma' of gallbladder. Gut (1975) 16(6):473–6. doi: 10.1136/gut.16.6.473 PMC1411036 168130

[4] Howlett TA Drury PL Perry L Doniach I Rees LH Besser GM . Diagnosis and management of ACTH-dependent Cushing's syndrome: comparison of the features in ectopic and pituitary ACTH production. Clin Endocrinol (Oxf) (1986) 24(6):699–713. doi: 10.1111/j.1365-2265.1986.tb01667.x 3024870

[5] Papastratis G Zografos GN Pappis HC Kontogeorgos G Anagnostopoulos G Kounadi T . ACTH-producing cholangiocarcinoma associated with Cushing’s Syndrome. Endocr Pathol (1999) 10(3):259–63. doi: 10.1007/BF02738888 12114708

[6] Lin D Suwantarat N Kwee S Miyashiro M . Cushing's syndrome caused by an ACTH-producing large cell neuroendocrine carcinoma of the gallbladder. World J Gastrointest Oncol (2010) 2(1):56–8. doi: 10.4251/wjgo.v2.i1.56 PMC2999155 21160818

[7] Lamberts SW Hofland LJ Nobels FR . Neuroendocrine tumor markers. Front Neuroendocrinol (2001) 22(4):309–39. doi: 10.1006/frne.2001.0218 11587555

[8] Liddle GW . Tests of pituitary-adrenal suppressibility in the diagnosis of Cushing's syndrome. J Clin Endocrinol Metab (1960) 20(12):1539–60. doi: 10.1210/jcem-20-12-1539 13761950

[9] Fleseriu M Auchus R Bancos I Ben-Shlomo A Bertherat J Biermasz NR . Consensus on diagnosis and management of Cushing's disease: a guideline update. Lancet Diabetes Endocrinol (2021) 9(12):847–75. doi: 10.1016/S2213-8587(21)00235-7 PMC8743006 34687601

[10] Raff H Carrol T . Cushing’s syndrome: from physiological principles to diagnosis and clinical care. J Physiol (2015) 593(3):493–506. doi: 10.1113/jphysiol.2014.282871 25480800 PMC4324701

[11] Wannachalee T Turcu AF Bancos I Amir Habra M Avram AM Chuang HH . The clinical impact of [^68^ Ga]-DOTATATE PET/CT for the diagnosis and management of ectopic adrenocorticotropic hormone - secreting tumors. Clin Endocrinol (Oxf) (2019) 91(2):288–94. doi: 10.1111/cen.14008 PMC6689243 31066920

[12] Mete O Wenig BM . Update from the 5th edition of the World Health Organization classification of head and neck tumors: overview of the 2022 WHO classifcation of head and neck neuroendocrine neoplasms. Head Neck Pathol (2022) 16(1):123–42. doi: 10.1007/s12105-022-01435-8 PMC9018952 35312985

[13] Kaltsas GA Besser GM Grossman AB . The diagnosis and medical management of advanced neuroendocrine tumors. Endocr Rev (2004) 25(3):458–511. doi: 10.1210/er.2003-0014 15180952

